# The impact of foot orthoses on gait in children with Osteogenesis Imperfecta type I, III and IV – a cross-sectional study

**DOI:** 10.1186/s12891-024-07672-y

**Published:** 2024-07-18

**Authors:** Josefine E. Naili, Eva Åström, Josefin Löwing, Mikael Reimeringer, Kristina Löwing

**Affiliations:** 1 Department of Women’s and Children’s Health, Karolinska Institutet, Karolinska Vägen 37 A, QA 02:07, 171 76 Stockholm, Sweden; 2 Motion Analysis Lab, Karolinska University Hospital, Stockholm, Sweden; 3 Department of Neuropediatrics, Astrid Lindgren Children’s Hospital, Karolinska University Hospital, Stockholm, Sweden; 4 Department of Women’s Health and Allied Health Professionals Theme, Medical Unit Occupational Therapy and Physiotherapy, Astrid Lindgren Children’s Hospital, Karolinska University Hospital, Gävlegatan 55, NB0:01, 171 76 Stockholm, Sweden

**Keywords:** Brittle bone, Genetic disorder, Physical rehabilitation, Walking, Barefoot, Orthotics, Insoles

## Abstract

**Background:**

For children with Osteogenesis Imperfecta (OI), a rare genetic bone disease, walking can be difficult to carry out due to a combination of bone fragility and deformity, muscle weakness, joint hypermobility, and pain. Bisphosphonate treatment has facilitated more children being able to walk, but for many, foot and ankle hypermobility is a limiting factor. Current evidence on foot orthoses in children with OI is sparse. This study aimed to evaluate gait characteristics in children with OI walking barefoot as compared to walking with foot orthoses.

**Methods:**

Twenty-three children with OI and hypermobility (mean age 8.3 ± 3.0 years) were included in this cross-sectional study. Children conducted three-dimensional gait analysis barefoot, and with foot orthoses and appropriate foot wear (stable yet light-weight), respectively. Walking speed, step length, lower limb kinematics and kinetics were collected. Differences in gait characteristics between test conditions were evaluated using paired sample t-tests.

**Results:**

When walking with foot orthoses, the external foot progression angle was reduced, peak ankle dorsiflexion angle increased, and peak plantarflexion moment increased as compared to barefoot. No difference was found in walking speed between test conditions, however, children with OI walked with longer steps with foot orthoses as compared to barefoot.

**Conclusion:**

The observed gait alterations suggest that foot orthoses, aiming to support the foot and ankle joint, contributed to reduced overall foot rotation as measured by external foot progression, increased peak plantarflexion moment, and increased step length. In a wider perspective, the ability to walk provides the opportunity to be physically active, and thereby increase skeletal loading and prevent fractures, thus, foot orthoses may be an important treatment option to consider in children with OI.

**Level of evidence:**

III.

## Introduction

Osteogenesis Imperfecta (OI) is a rare genetic disorder characterized by osteopenia and bone fractures, as well as extra-skeletal manifestations, such as dental abnormalities, blue sclera, hearing loss and joint hypermobility [1]. In a majority of affected individuals, it is caused by dominant inherited pathogenic variants in the genes coding for the collagen (COL1A1 or COL1A2) [1]. The global incidence is around 1/10–20 000 births [2]. The Sillence classification has been used for many years, and is based on the clinical phenotype, with type I being the less severe, type II lethal perinatally or soon after birth, type III the most severe type of OI associated with survival of the neonatal period, and type IV of intermediary severity [3]. However, today the genome database Online Mendelian Inheritance in Man (OMIM) includes 22 types of OI (Type I-XXII).

Today infants with moderate and severe phenotypes of OI receive intravenous bisphosphonate treatment which support motor development, reduce pain, and increase bone density, as well as facilitating for these children being able to walk [4–6]. Previously reported important aspects of quality of life in children with OI include reduced functioning, independence, and pain [7]. Mobility, including ambulatory capacity, can affect each of these themes and are important for children with OI [8]. In some children with OI, hypermobility can be the most limiting factor for physical activity, and particularly walking capacity [9]. Hypermobility is often present in the foot and ankle joint and can sometimes, over time, develop into excessive hind-foot valgus, a collapsed longitudinal arch, and forefoot abduction, contributing to increased risk of falling, muscle weakness, misalignment, and pain, all of which in the long run can lead to walking difficulties and thereby reduce physical activity [10, 11]. A vicious circle of inactivity may begin, if children with OI are not provided with the means to be physically active, which further decreases bone density and increase the risk of fractures [12, 13].

Previous litterature suggest that in patients with connective tissues disorders, such as Ehlers Danlos syndrome, Downs syndrome, and OI, foot orthoses may improve pain and fatigue [14], as well as stability [15]. Arch supporting foot orthoses have been shown to decrease the external ankle evertor moment in children with flexible flat foot deformity [16], and to reduce variability in spatiotemporal gait parameters in children with joint hypermobility syndrome [17]. In children with juvenile idiopathic arthritis, foot orthoses, as compared to shoes only, have been reported to reduce pain and improve function [18], and redrestribute high pressure areas of the hind-foot and forefoot [19]. A recent review, evaluating foot orthoses for treating pediatric flat feet, concluded that attention should be directed towards pediatric foot conditions causing pain, limited function, or reduced quality of life [18], all of which are common difficulties for children with OI [7, 8, 20].

This study aimed to evaluate the impact of foot orthoses and shoes on gait patterns and spatiotemporal parameters in children with OI and ankle/foot hypermobility. It was hypothesized that shoes and orthoses during gait would alter the foot position and contribute to reduced overall foot rotation. Secondly, it was hypothesized that foot orthoses would lead to increased walking speed.

## Materials and methods

### Study design and study participants

Ethical approval for this cross-sectional study was obtained from Stockholm’s regional ethical review board (DNR: 2017/1136–31/2). The STROBE guidelines were followed in the development and reporting of this study [21]. All participants and their caregivers received oral and written information about the study procedures. Children provided verbal informed assent, and caregivers provided verbal and written informed consent prior to study participation in accordance with the Declaration of Helsinki. Inclusion of study participants began in May 2017 and ended in February 2021. A priori sample size estimations were performed on a sub-set of data of 15 children with OI. To detect a difference of 5 degrees’ external foot progression, and a difference of 0.1 m/s in walking speed, with the power set at 0.8, 22 children were needed to detect a difference in foot progression angle, and 10 individuals to detect a difference in walking speed.

### Recruitment process

**Fig. 1:**
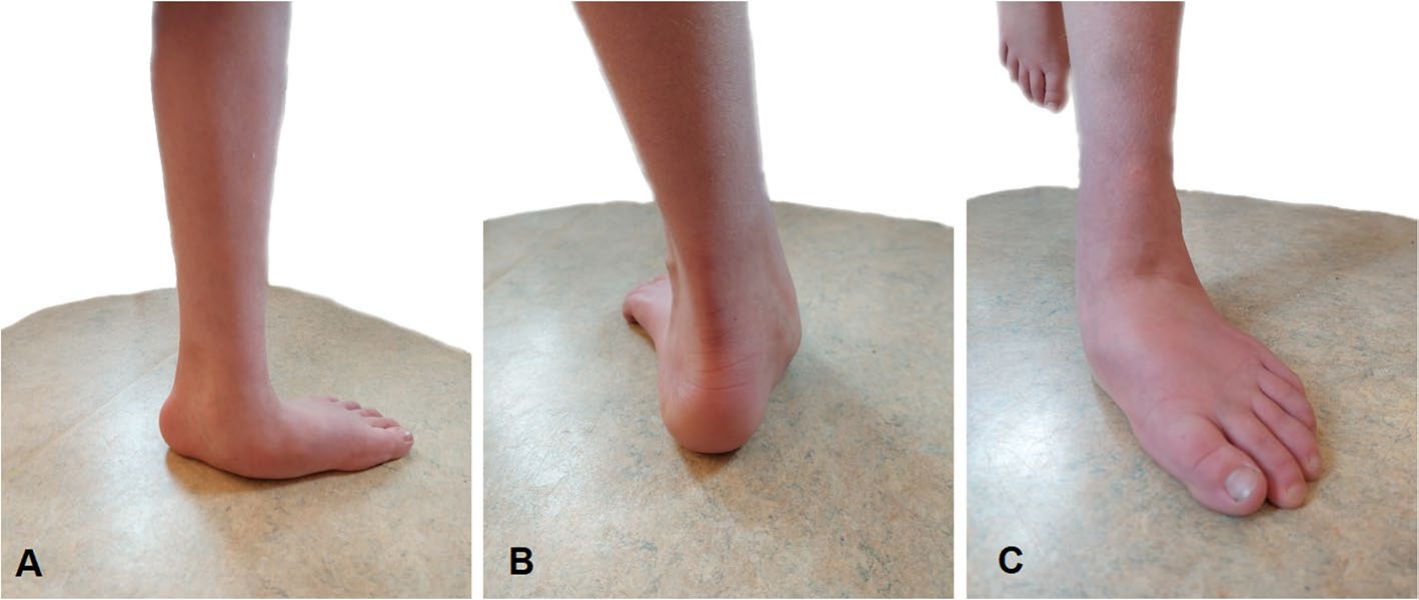
Example of a child with OI type I and joint hypermobility. **A** Left foot seen in a sagittal view of the medial side of the foot with a collapsed longitudinal arch. **B** Left foot seen in a posterior view of the hind-foot with increased valgus alignment. **C** Left foot seen in an anterior view of the forefoot with increased abduction

**Fig. 2:**
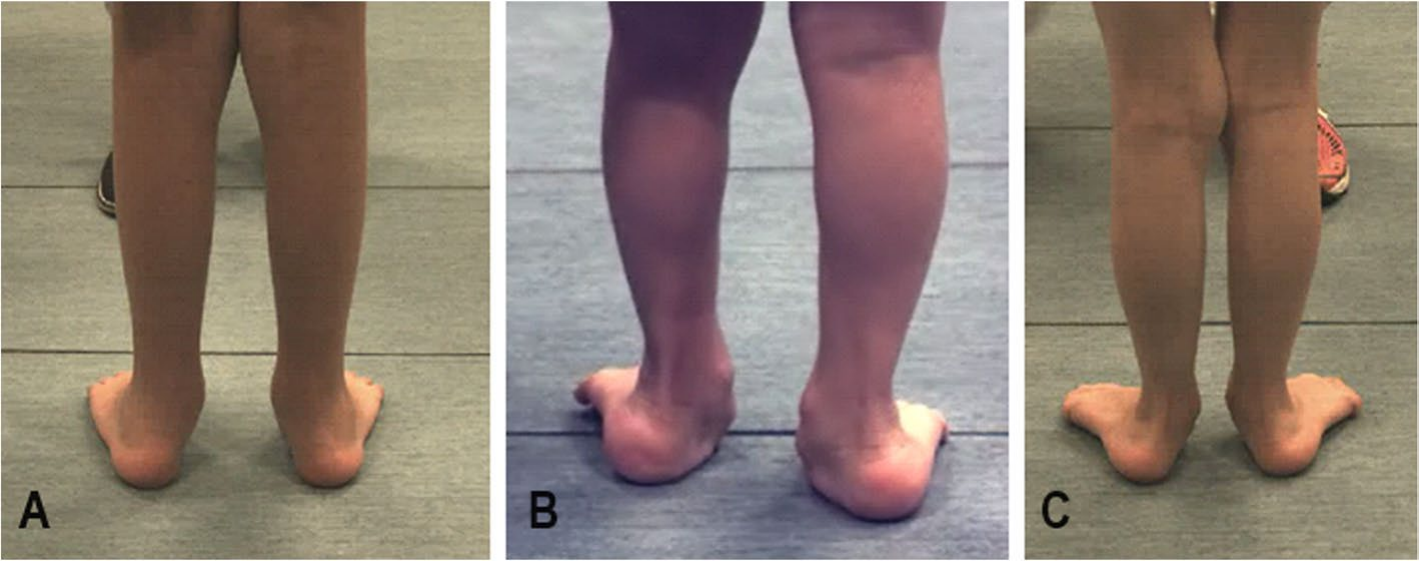
Posterior view of **A**) A child with Osteogensis Imperfecta (OI) type I that was prescribed customized insoles. **B** A child with OI type III that was prescribed customized insoles. **C** A child with OI type I prescribed a supra-malleolar orthosis

In the present study, children with OI, with clinical hypermobility and walking ability was recruited consecutively at their regular clinical OI team visit at Astrid Lindgren Children’s Hospital, Karolinska University Hospital, Stockholm, Sweden. This is a tertiary children’s hospital, and the national center for children with OI, at which children with OI are regularly examined by the specialists within the OI team. The team consists of neuro pediatrician, physiotherapist, occupational therapist, orthopedic surgeon, nurse, dentist, and certified prosthetists and orthotists (CPO). A strict consecutive inclusion of study participants was not possible due to logistic challenges coordinating clinical evaluations during team visits including intravenous bisphosphonate treatment, bone density evaluation, radiological assessments, and appointments with health care professionals. The inclusion criteria were children with OI and hypermobility in the lower-extremity joints (Figs. 1 and 2), prescribed customized foot orthoses, ability to walk 10 m repeatedly without the use of a walking aid, and the ability to communicate verbally (and in writing for caregivers) in Swedish. Exclusion criteria included fractures in the lower extremity and/or spine within 6 months. Presence of hypermobility was assessed during physical examinations and included passive range of motion measured using a goniometer, inspection of foot position at rest (without weight-bearing) and in standing (Table 2), and through observation in motion (i.e. during walking). From a posterior view, the position of the calcaneus and the number of toes that deviated laterally in standing were observed (Table 2). The most common locations of hypermobility for the included children with OI included knee, ankle, foot, and finger joints (Figs. 1 and 2).

### Foot orthoses

**Fig. 3:**
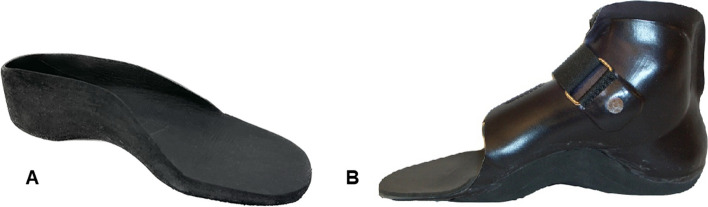
Examples of foot orthoses prescribed for children with Osteogenesis Imperfecta. **A** A customized insole made out ethylene–vinyl acetate foam. **B** A supra-malleolar orthoses made of 2 mm polypropylene co-polymer with a thin lining of leather or heat moldable foam

Within the OI team, foot orthoses are prescribed by the OI team physician when children present with the hind-foot in valgus to the degree where the foot starts to tip medially and when children/caregivers report ankle instability (i.e. complaints of repeated ankle sprains, or impaired balance). For children with more severe foot deformity (i.e. excessive hind-foot valgus, a collapsed longitudinal arch, forefoot abduction and reduced floor contact of the lateral border of the foot), supra-malleolar orthoses (SMO) are prescribed (Fig. 3). Children with SMO have usually previously tried out customized insoles, but deemed in need of additional support. Children presenting with flat foot only are not prescribed foot orthoses.

The included children came from different regions in Sweden and the custom insoles were made in their home-region. Two impression techniques used were utilized; casting or scanning of the children's feet, to create a model for the custom-made insoles. The choice of method was determined by the CPO’s preference and experience. Insoles were made of ethylene–vinyl acetate (EVA)-foam (shore ranging between 35–55) (Fig. 3). To create a model for the SMO, a circular plaster cast of the feet was made. The SMO was made of 2 mm polypropylene co-polymer (PPC) with a thin lining of leather or heat moldable foam. Both EVA and PPC was molded with vacuum technique over the model to achieve a close-fitting orthosis (Fig. 3). All foot orthoses were carefully adapted by the CPO at their local region for health care, with support from the CPO in the OI team if needed.

In the present study, all of the included children had worn prescribed customized foot orthoses for at least one year. Twenty children had bilateral customized insoles, and three children had bilateral customized supra-malleolar orthoses (SMO) (out of which two children had OI type I, and one child had OI type III) (Fig. 2) (ISO 061203) [22, 23].

### Three-dimensional gait analysis

Three-dimensional (3D) gait analysis was performed at one occasion at the Motion Analysis Lab, Astrid Lindgren Children’s Hospital, Karolinska University Hospital, Stockholm, Sweden. The Motion Analysis Lab was equipped with an eight-camera system, Vicon MX (sampling rate 100 Hz) (VICON, Oxford, UK) and two staggered Kistler force plates embedded in the floor (Kistler, Winterthur, Switzerland).

Each child was examined with 3D gait analysis when walking barefoot and then when walking with the foot orthoses and shoes they used in their everyday life, which meant that each child was her/his own control.

Each test session started with a physical examination where passive range of motion of lower extremity joints, foot-thigh-angle, and bimalleolar axis were measured with a goniometer with the patient lying on an examination table (Table 2). Anthropometric measures of height, weight, leg length, and joint width were measured. Through inspection, foot position at rest and in standing was assessed, and presence of lower-limb deformity noted (Table 2). Following the physical examination, children were asked to stand with their feet placed on two horizontal lines marked by tape on the floor. Participants stood with the long axis of each foot on these lines; both when markers were placed on bare feet, and later on when markers were placed on shoes (including foot orthoses). The participants had a frame placed in front of them for balance support. Sixteen reflective markers of nine mm diameters were placed on predefined standardized anatomical landmarks according to a conventional biomechanical gait model (Plug-In-Gait) [24, 25]. Good intra-sessional repeatability has been reported using this model [26]. Markers were placed by the one and same experienced physiotherapist at all assessments. Prior to placing markers during the test condition with foot orthoses and shoes, ankle joint width measures were re-taken. The physiotherapist placing the markers had the horizontal line on the floor as a reference point, as well as other bony landmarks and anatomical structures to palpate (fibula head, Achilles tendon, lateral/medial malleolus depending on type of shoes).

Children were instructed to walk barefoot first, and following with the foot orthoses and shoes they use in their everyday life, repeatedly along a defined 10-m pathway at a self-selected walking speed. Recordings were made in two directions (back and forth). Kinematic, kinetic, and time and distance data were collected simultaneously. Kinetics were expressed by internal moments normalized to body weight. Each test session took around 90 min to complete.

### Study participant characteristics

Information regarding type of OI, recent fractures, and total number of fractures was retrieved from the patients’ medical records. Children were asked about the presence of pain (yes/no), and maximal walking distance (in meters) was estimated by children with support from their parents. This information was obtained by the physiotherapist in the OI team at the time for the gait analysis. Presence of lower-limb deformity (yes/no) was determined based on radiographic examination (Table 2).

### Data analysis

Gait data was processed using Vicon Nexus software 2.9.1 (Vicon Motion Systems Ltd, Oxford, UK), and raw motion capture data was filtered using a Woltring filter set to 20, mode mean squared error (MSE). Hypermobility in the foot and ankle may lead to excessive hind-foot valgus, a collapsed longitudinal arch, and forefoot abduction (Figs. 1 and 2) [27]. In this position, dorsiflexion range of motion is not necessarily utilized in the talocrural joint, but the mid-tarsal joint [28, 29]. The lever arm is shortened, and stretch of the calf-muscle during terminal stance is reduced [30], which may contribute to reduced plantarflexion moment and an inefficient gait pattern [31]. Thus, to assess the impact of foot orthoses on gait, as compared to barefoot, the kinematic variables of interest included peak dorsiflexion angle, peak plantarflexion angle, knee angle at initial contact, peak knee flexion angle, peak knee extension angle, and peak external foot progression angle (the foot relative to the global coordinate system of the laboratory). Pelvic rotation range and peak external hip rotation were also evaluated. Kinetic variables of interest included peak plantarflexion moment, peak knee flexion moment, peak knee extension moment. The sign conventions used in the present study adopt knee flexion, ankle dorsiflexion, internal foot progression, and internal hip rotation as positive motions, and knee extension and plantarflexion moments as positive. Time and distance data included walking speed, step length and step width. Step length was normalized by leg length, self-selected walking speed (velocity from heel strike to subsequent heel strike) and step cadence (steps/minute) were normalized by leg length and gravity as described by Hof [32].

**Fig. 4:**
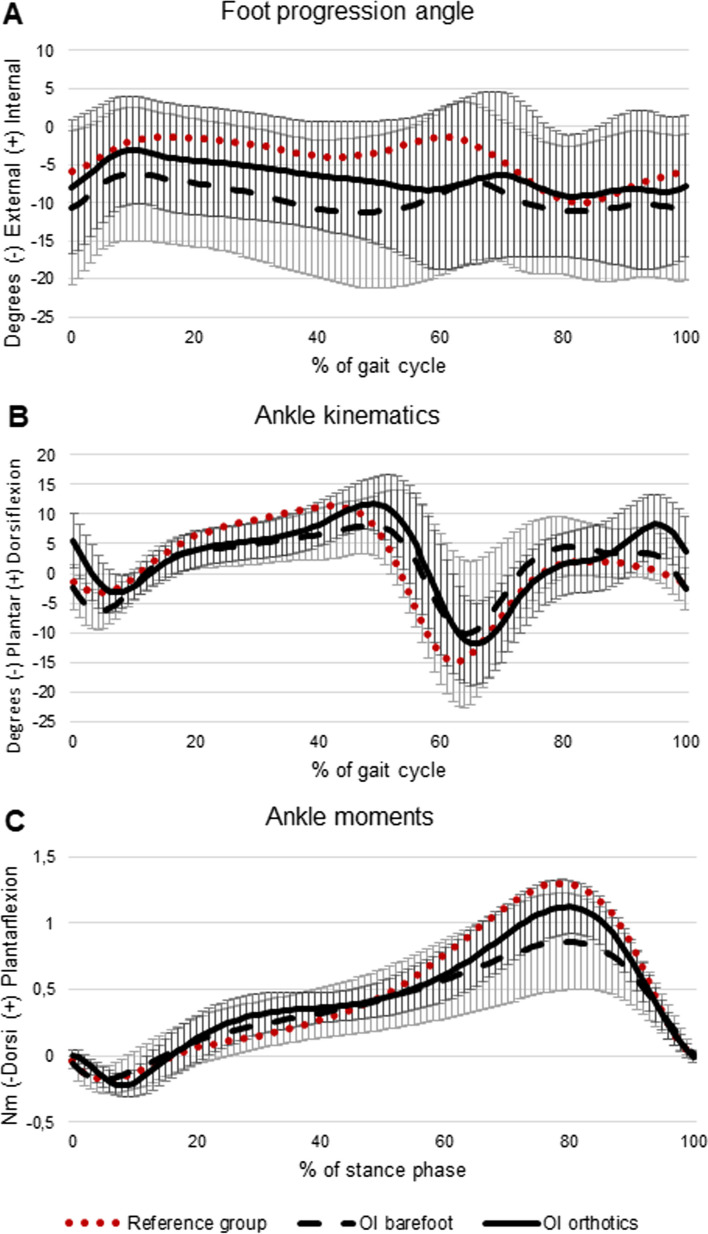
Foot progression angles (*n* = 23) (**A**), sagittal plane joint angles (*n* = 23) (**B**) and sagittal plane joint moments (*n* = 18) (**B**) in children with Osteogenesis Imperfecta (OI) walking barefoot, and with foot orthoses, respectively. The reference group consists of 18 typically developing children with a mean age of 9 years

**Fig. 5:**
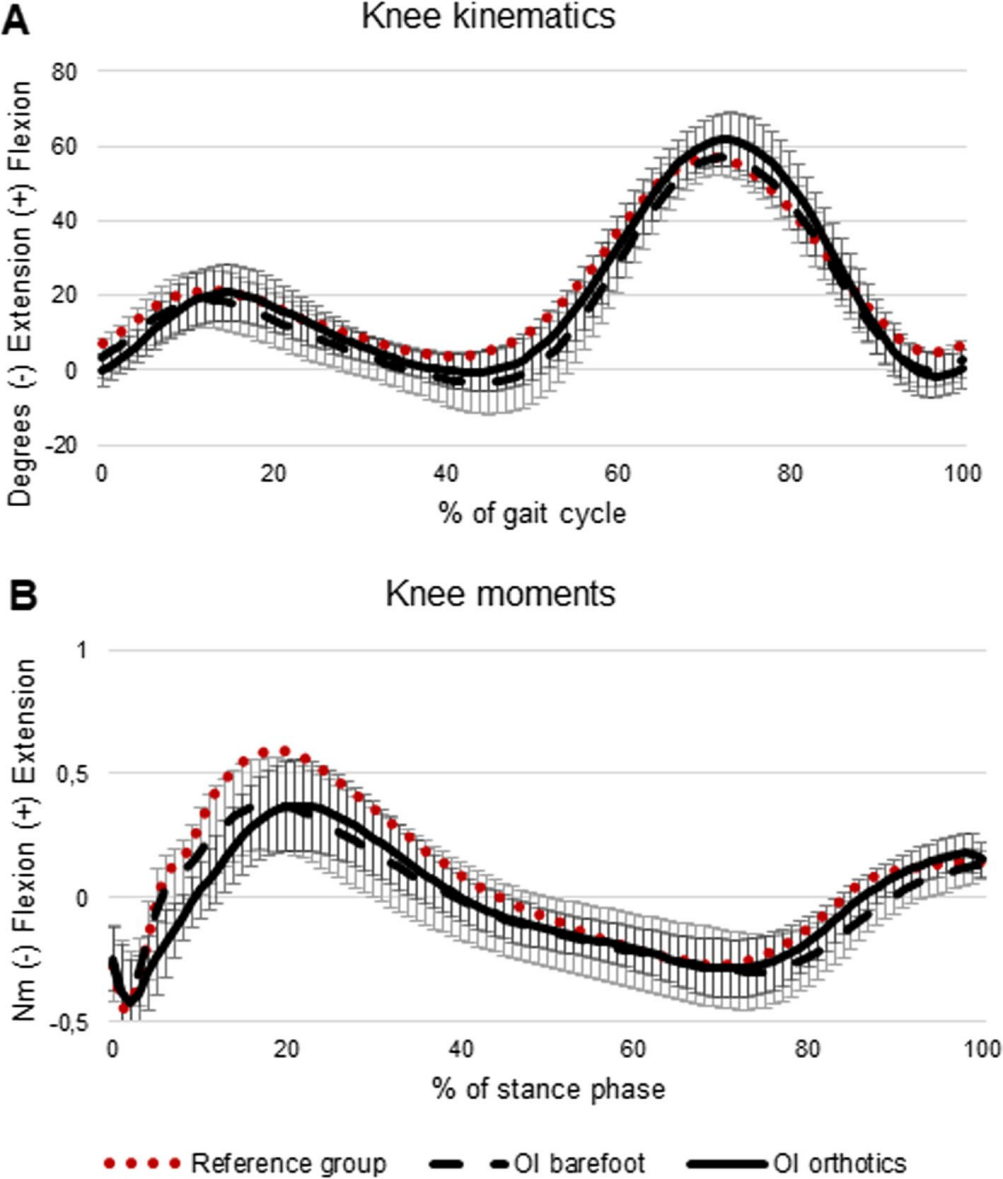
Sagittal plane knee joint angles (*n* = 23) (**A**) and knee joint moments (*n* = 18) (**B**) in children with Osteogenesis Imperfecta (OI) walking barefoot, and with foot orthoses, respectively. The reference group consists of 18 typically developing children with a mean age of 9 years

At least three gait cycles with good marker visibility, clean force plate strikes, and consistent walking speed were analyzed for each patient, for each test condition (barefoot vs. with foot orthoses). In five children with OI, no clean force plate strikes were obtained due to short step length. For these children, only kinematic and time- and distance data were included in the analysis. The included children with OI presented with symmetrical gait data, i.e. no significant differences between the right or left side. Since walking is a bilateral activity, including two legs that are not independent from each other, considering limbs separately leads to larger number of data points and increased power of the statistical calculations [33]. Thus, we arbitrarily chose to include the right side [33]. For each participant, and for each test condition, data derived from the included gait cycles were averaged. Calculations of gait data were performed using MATLAB® software R2016b (The MathWorks, Inc, Natick, MA). To facilitate valuation of impact of the disease on gait biomechanics, ankle kinematics and kinetics of the studied group of children with OI were graphically illustrated alongside an age-matched reference group of typically developing children from the control database recorded at the same gait lab (Motion analysis lab at Astrid Lindgren Children’s Hospital, Karolinska University Hospital) (Figs. 4 and 5). The reference group consisted of 18 children with a mean age of 9.1 ± 2.4 (range 5–14) years.

### Statistical analysis

All data were analyzed using SPSS statistics (IBM, Version 26 Armonk, NY, USA). The statistical significance level was set at ɑ = 0.05. Normality of data was assessed with Shapiro Wilk’s test and Q-Q plots. Descriptive data were reported as mean, standard deviation, median, range, and frequency. Differences between test conditions in continuous gait data were analyzed using a parametric Student’s t-test. To evaluate the magnitude of differences between test conditions, effect sizes (Cohen’s *d*) were calculated [34], and interpreted as small (d = 0.2), medium (d = 0.5), and large (d = 0.8) [35]. To evaluate the differences between children with clean force plate data (joint kinetics) and children with no clean force plate data, independent sample t-tests were carried out to compare study participant characteristics (i.e. age, body height, leg length).

## Results

### Participant characteristics and patient-reported outcomes

Twenty-three children with OI, 16 boys and 7 girls, with a mean age of 8.3 ± 3.0 (range 5–16) years were included in the study. OI type I was the dominating type of the included children, and median number of previous fractures was 8 (range 2–50) (Table 1). Twelve children (out of 23) received bisphosphonate treatment. Pain was present in 20 out of 23 children, the patient-reported maximal walking distance varied between 25 m and 10 km, and 23 out of 23 children reported daily wear of foot orthoses (Table 1). All of the included children presented with forefoot abduction in standing, with no toes visible medially, and the vast majority of children presented with hind-foot valgus in standing (Table 2).

**Table 1:** Study participant characteristics of included children with osteogenesis imperfecta

Study participants	
n (Girls)	23 (7)
Age, years	
Mean (SD)	8.3 (2.9)
Range	5–16
Body height (cm)	
Total group mean (SD)	124 (20)
Girls mean (SD)	109 (12)
Boys mean	130 (19)
Body Mass Index	
Total group mean (SD)	16.4 (2.9)
Girl mean (SD)	15.9 (1.1)
Boy mean (SD)	16.5 (3.5)
Osteogenesis Imperfecta type (n)	
I	17
III	3
IV	3
Bisphosphonate treatment (n)	
Yes	12
No	11
Current total number of fractures	
1–10	16
11–20	4
21–30	1
31–50	2
Most recent fracture/surgery (n)	
Foot	3
Tibia	6
Femur	3
Spine	6
Upper extremity	5
Intramedullary nailing (n)	
None	13
Unilateral Tibia	2
Bilateral Tibia	3
Unilateral Femur	2
Bilateral Femur	5
Upper extremity	2
Reported use of foot orthoses (n)	
Daily	23
Maximal walking distance (n)	
25 m	1
0.2—0.5 km	4
0.6—1 km	4
2—5 km	11
6—10 km	3
Musculoskeletal pain (n)	
Yes	20
No	3

*SD* Standard Deviation

**Table 2:** Physical examination of included children with Ostoegenesis Imperfecta at the time for gait analysis

ID	OI type	Passive range of motion right limb (degrees)						Lower-limb deformity		Foot position at rest (without weight bearing)		Foot position in standing		
		**Hip**		**Knee**		**Ankle**		**Femur**	**Tibia/Fibula**	**Pes planus**	**Hind-foot valgus**	**Pes planus**	**Hind-foot valgus**	**Forefoot abduction**^a^
		**Flexion**	**Extension**	**Flexion**	**Extension**	**Dorsiflexion (flexed knee)**	**Plantarflexion**							
**1**	I	130	15	155	15	25	40	No	No	Yes	Yes	Yes	Yes	Bilateral
**2**	III	120	10	150	20	15	45	Yes	Yes	No	No	Yes	Yes	Bilateral
**3**	I	120	15	155	10	30	60	No	No	No	No	Yes	Yes	Bilateral
**4**	I	120	15	155	20	30	60	No	No	No	No	Yes	Yes	Bilateral
**5**	I	130	10	155	20	30	60	No	No	No	No	Yes	Yes	No
**6**	I	120	20	155	15	30	60	No	No	No	No	Yes	Yes	Bilateral
**7**	I	120	20	155	5	30	60	No	No	No	No	Yes	Yes	Bilateral
**8**	I	125	15	155	10	25	60	No	No	No	No	Yes	Yes	Bilateral
**9**	IV	130	20	155	15	25	60	No	No	No	No	Yes	Yes	Bilateral
**10**	III	120	10	150	0	10	55	Yes	Yes	No	No	Yes	Yes	Bilateral
**11**	I	130	10	155	15	20	60	Yes	Yes	Yes	Yes	Yes	Yes	Bilateral
**12**	III	120	20	155	10	30	60	Yes	Yes	No	No	Yes	Yes	Bilateral
**13**	I	130	15	155	15	10	60	No	No	Yes	Yes	Yes	Yes	Bilateral
**14**	I	130	15	155	10	30	60	No	No	No	No	Yes	Yes	Bilateral
**15**	I	130	20	155	20	10	50	No	No	No	No	Yes	Yes	Bilateral
**16**	IV	130	15	155	15	30	40	No	No	No	No	Yes	Yes	Bilateral
**17**	I	130	15	155	20	30	50	No	No	No	No	Yes	Yes	Bilateral
**18**	I	125	15	155	10	35	60	No	No	No	No	Yes	No	Bilateral
**19**	I	120	15	150	5	20	40	No	No	No	No	Yes	Yes	No
**20**	I	110	20	155	10	25	50	No	No	No	No	Yes	Yes	Bilateral
**21**	I	125	20	155	5	10	45	No	No	No	No	Yes	Yes	Bilateral
**22**	IV	130	15	155	0	30	35	No	No	No	No	Yes	No	Bilateral
**23**	I	130	15	155	20	20	60	No	No	Yes	Yes	Yes	Yes	Bilateral

^a^Forefoot abduction, at least three toes visible laterally

### Ankle kinematics and kinetics during gait

With foot orthoses, children with OI displayed less external foot progression, increased peak ankle dorsiflexion angle, and increased peak plantarflexion moment as compared to barefoot (Table 3, Fig. 4).

**Table 3:** Self-selected walking speed, step length, lower limb joint kinematics and kinetics (expressed as internal moments) during level gait among included children with Osteogenesis Imperfecta. Children were walking barefoot and with foot orthoses, respectively

	**Barefoot**	**Foot orthoses**	**Effect size**	***p*-value**
	**(*n* = 23)**			
**Time and distance data**	Mean (SD)			
Walking speed (m/s)	1.0 (0.18)	1.1 (0.23)	0.48	0.119
Normalized walking speed	0.42 (0.07)	0.44 (0.09)	0.25	0.118
Step length (m)	0.38 (0.18)	0.44 (0.16)	0.35	**< 0.001**
Normalized step length	0.60 (0.26)	0.70 (0.28)	0.33	**< 0.001**
Step width (m)	0.13 (0.03)	0.12 (0.04)	0.28	0.056
**Kinematics (degrees)**				
**Ankle**				
Peak ankle dorsiflexion ( +)	13.1 (5.1)	15.7 (5.1)	0.51	**0.007**
Peak ankle plantarflexion (-)	-13.3 (7.7)	-12.9 (7.8)	0.05	0.719
Peak external (-) foot progression	-20.3 (11.4)	-14.2 (8.7)	0.60	**0.001**
**Knee**				
Knee angle at initial contact	0.4 (6.3)	-1.7 (7.0)	0.30	0.059
Peak knee flexion ( +)	58.1 (4.8)	62.7 (6.9)	0.77	**0.008**
Peak knee extension (-)	-6.2 (7.1)	-5.3 (3.9)		0.610
**Hip**				
Peak external (-) hip rotation	-14.4 (6.8)	-16.1 (8.2)	0.16	0.195
**Pelvis**				
Pelvic rotation range	20.6 (9.1)	19.5 (8.4)	0.12	0.477
**Kinetics (Nm/bodyweight)**	**Barefoot**	**Foot orthoses**	**Effect size**	***p*-value**
	**(*n* = 18)**			
**Ankle**				
Peak plantarflexion moment ( +)	0.93 (0.29)	1.14 (0.19)	0.82	**0.004**
**Knee**				
Peak flexion moment (-)	-0.48 (0.17)	-0.48 (0.18)	0	0.897
Peak extension moment ( +)	0.49 (0.17)	0.46 (0.17)	0.18	0.886

*Nm* Newton meter, *SD* Standard Deviation

### Knee kinematics and kinetics during gait

Peak knee flexion angle during swing increased when walking with foot orthoses as compared to barefoot (Table 3, Fig. 5). No differences were found in sagittal plane knee joint moments between test conditions (Table 3).

### Pelvis and hip kinematics during gait

No differences were found in pelvic rotation range or peak hip rotation between test conditions (Table 3).

### Time and distance data

With foot orthoses, children with OI walked with longer steps as compared to barefoot however, with unchanged walking speed and step width (Table 3).

### Missing data

The five children with no clean force plate strikes, and thus excluded from the analysis of joint kinetics, were significantly younger (mean 6 vs. 9 years, *p* = 0.027), shorter (mean 106 vs. 128 cm, *p* = 0.021) and had shorter legs (mean 52 vs 67 cm, *p* = 0.018) as compared to the remaining group of children.

## Discussion

This cross-sectional study aimed to evaluate the impact of foot orthoses on gait characteristics in children with OI. To this end, differences in gait dynamics and spatiotemporal parameters were evaluated and compared between walking barefoot and with foot orthoses and appropriate foot wear, respectively. It was hypothesized that foot orthoses during gait would alter the foot position and contribute to reduced overall foot rotation as measured by an external foot progression angle, which would lead to a longer lever arm, redistribution of dorsiflexion to the talocrural joint [28, 29], increased stretch of the calf-muscle during terminal stance, and increased walking speed [27]. The observed differences between test conditions suggest that foot orthoses contributed to reduced external foot progression. With foot orthoses, increased ankle dorsiflexion during stance, increased peak plantarflexion moment, increased knee flexion during swing, as well as increased step length was also observed compared to barefoot. The increased knee flexion could be related to increased step length, but also to increased demands for foot clearance as compared to barefoot.

In the present study, gait patterns of children with OI were compared graphically to an age-matched reference group of typically developing children (Figs. 4 and 5). The gait deviations observed are in accordance with previous research evaluating gait patterns in children with OI including reduced overall ankle range of motion during stance, longer stance phase duration, and reduced peak ankle push off power (generation) [36, 37]; reduced walking speed and step length [10, 37]. Furthermore, overall kinematic gait deviations (i.e. kinematic waveform analysis) were evaluated using a comprehensive summary measure, the Gait Deviation Index (GDI), which demonstrated lower GDI scores of children with OI as compared to controls [10]. Taken together, previous studies and the current study demonstrate that children with OI walk with reduced spatiotemporal gait parameters, as well as with gait pattern deviations across multiple joints as compared to age-matched controls. To further quantify the severity of forefoot abduction and hind foot valgus, future research should consider utilizing a biomechanical model including a multi-segment foot model for evaluation of barefoot gait dynamics [38]. Waveform analysis, to assess both magnitude and temporal effects on biomechanical data, could further inform the deviant gait characteristics [39].

The root of gait deviations observed in children with OI are multi-facetted. Deviations may be related to bone deformity, hypermobility [1], reduced muscle strength [40], and pathogenic variants affecting collagen [41]. The severe phenotypes of OI lead to progressive bone deformity, poor bone healing and growth delays [42, 43]. Furthermore, the severity of OI has a large influence on the age and sequence in the development of motor milestones [44]. However, today infants with moderate and severe phenotypes of OI receive intravenous bisphosphonate treatment, already before the age of one year, which support motor development, reduce pain, and increase bone density [4–6]. Results of intravenous bisphosphonate treatment has demonstrated that children with moderate and severe phenotypes have the possibility to walk [4–6]. A recent study by Montpetit et al., reported that independent ambulatory function acquired before five to six years of age was the clinical characteristic that could predict maintained ambulatory function at around 13 years of age in children with OI [8]. Thus, a cornerstone in OI treatment is to facilitate for children to walk before the age of around five years [8].

Pes planus (flatfoot) is a common pediatric condition, generally resolved during adolescence, with greatly varied prevalence estimates [18]. In children with flatfoot *accompanied by pain*, treatment options include foot orthoses or shoe inserts, muscle stretching, footwear selection, physical activity modification, and reducing body weight [18]. As outlined throughout this study, foot deformity is not the sole negative influence on gait in children with OI, as the condition also involves muscle weakness, joint hypermobility, and malalignment of the of the skeleton [1, 40, 42, 43]. In a recent cross-sectional study exploring pain and pain interference in children with OI, with a partly overlapping cohort as the present study (*n* = 5), the most common cause of non-fracture pain explained by children was “walking for longer distances” [20]. In a retrospective cohort study, Adib et al. defined clinical characteristics of 124 children (median age of 12 years) with joint hypermobility-related presentations [45]. The major complaints were joint pain (74%), atypical gait (10%), joint deformity (10%), and back pain (6%). Close to half of these children (48%) had major limitations of school-based physical education activities, and two thirds of the cohort (67%) limitations of other physical activities [45]. In summary, it can be concluded that both pain and joint hypermobility are two factors to consider when planning treatment aiming to increase physical activity levels in children with OI.

The present study has several limitations. Firstly, inclusion of study participants was not carried out in a consecutive manner. The current study sample consists of children for whom additional assessments were possible to fit into an already busy schedule of hospital appointments during team visits, in addition to the specific inclusion and exclusion criteria. Consequently, the studied group may be subjected to selection bias. The busy schedule of appointments also affected the choice to not randomize the order of test conditions; barefoot or with foot orthoses, and not to have a third test condition evaluating only foot wear which would have been valuable in discerning the effect of foot orthoses compared to shoes only. To provide space for the foot orthoses, the children were required to have a larger shoe size. If the insoles were removed, the shoes became too big. Thus, the shoes worn by the children were not appropriate in terms of being close-fitting when the foot orthoses were removed. Since each gait lab assessment started with a physical examination barefoot, a decision was made to perform all barefoot testing first, to avoid additional shoes on and off. Thus, for the gait trials with foot orthoses, children may have started to feel tired from performing multiple walking trials, which may have impacted the results. Secondly, placing markers on shoes poses many challenges, as some of the bony landmarks where markers are placed cannot be seen or palpated through a shoe. Measures were taken to standardize carful marker placement by having participants stand on marked lines and carful palpation of other bony landmarks visible, however, absolute accuracy cannot be guaranteed. Thirdly, the included children with OI had different types of OI, where type I dominated the cohort. This has an impact on the generalizability of the results.

The strengths of the present study include a relatively small, however, sufficient sample size of children with a rare genetic disorder. All gait analyses and related examinations were carried out by two experienced physiotherapists. Gait analyses of both test conditions (barefoot vs. foot orthoses) were performed during one session, limiting the potential effects of fluctuations in the included children’s state of health. The statistically significant differences observed are not necessarily clinically relevant, however, when accompanied by measures of effect size the magnitude of differences between test conditions may facilitate interpretation. Results of the present study should be interpreted in a wider clinical perspective together with reported use of foot orthoses, perceived pain, and activity level.

## Conclusion

The observed gait alterations suggest that foot orthoses, aiming to support the foot and ankle joint, contributed to reduced overall foot rotation as measured by external foot progression, increased peak plantarflexion moment, and increased step length as compared to walking barefoot. In a wider perspective, the ability to walk provides the opportunity to be physically active on a daily basis, and thereby increase skeletal loading, and hopefully prevent pain and fractures. Foot orthoses may therefore be an important treatment option in children with OI.

## Data Availability

The datasets used and analyzed during the current study are not publicly available due to ethical concerns, but are available from the corresponding author on reasonable request.

## Abbreviations

CPO: Certified prosthetists and orthotists
EVA: Ethylene–vinyl acetate
GDI: Gait Deviation Index
MSE: Mean squared error
OI: Osteogenesis Imperfecta
OMIM: Online Mendelian Inheritance in Man
PPC: Polypropylene co-polymer
SD: Standard Deviation
SMO: Supra-malleolar orthoses
3D: Three-dimensional
